# Fahr’s Syndrome in a Greenlandic Inuit

**DOI:** 10.5334/jbr-btr.1439

**Published:** 2018-01-04

**Authors:** Anne Toftgaard Pedersen, Ida Kledal, Luit Penninga

**Affiliations:** 1 Ilulissat Regional Hospital, Ilulissat, Greenland

**Keywords:** Fahr’s disease, Fahr’s syndrome, brain calcification, brain imaging, CT-scanning

Fahr’s disease or syndrome is a rare inherited or sporadic neurological disorder with a prevalence of less than 1/1,000,000 [1]. Recently, a number of genes have been identified, known to be associated with familial primary brain calcification (PFBC), causing Fahr’s disease or syndrome [1]. These genes are SCL20A2, PDGFB, PDGFRB and XPR1, and a significant correlation was found between the presence of headache in PDGFB mutations, and parkinsonism with SLC20A2 [2].

**Figure 1:**
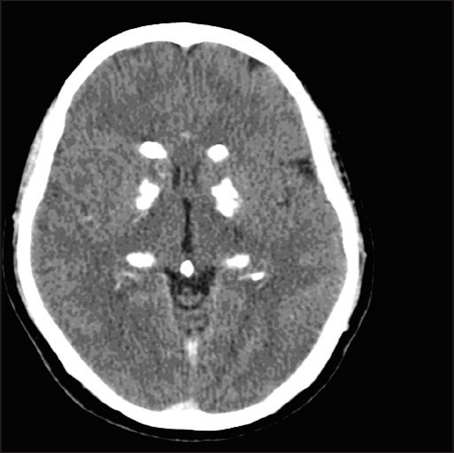
CT scan of the brain: Transverse view shows bilateral calcifications of the thalamus and corpus striatum.

**Figure 2:**
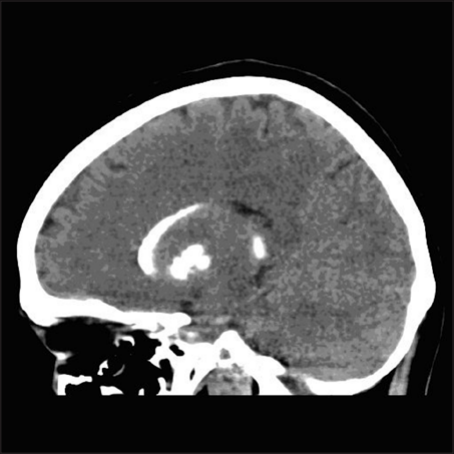
CT scan of the brain: Sagittal plane shows calcifications of the thalamus and corpus striatum.

We present the case of a 49-year-old woman with Greenlandic Inuit origin and an otherwise unremarkable medical history. She initially presented with symptoms of headache and diplopia. She was referred to a CT scan of the cerebrum, which showed bilateral calcifications of the thalamus and corpus striatum (Figures 1 and 2) compatible with Fahr’s syndrome [1]. Shortly after, the patient developed tremor of the right hand, fatigability especially of her right side, dizziness, and minor cognitive decline.

Laboratory studies showed no metabolic disorders such as hypopituitarism, parathyroid disturbances or significant changes in the calcium-phosphate metabolism, besides a minimal hypocalcaemia (free calcium: 1.12 mmol/L, reference 1.17–1.34 mmol/L), which was interpreted as secondary to D-vitamin deficiency (13 nmol/L), very common in the arctic region.

No other primary cause for brain calcification was detected, and the patient has a younger sister, who is also diagnosed with Fahr’s syndrome, suggestive of an inherited type.

The present case shows that symptoms of Fahr’s syndrome may be few. Furthermore, this exemplifies that neurological manifestations may be confined to one side of the body, despite the presence of bilateral calcifications [1].

This case illustrates the importance of neuroimaging for establishing the diagnosis.
